# Method comparison of HPLC-ninhydrin-photometry and UHPLC-PITC-tandem mass spectrometry for serum amino acid analyses in patients with complex congenital heart disease and controls

**DOI:** 10.1007/s11306-020-01741-8

**Published:** 2020-12-15

**Authors:** Miriam Michel, Christina Salvador, Verena Wiedemair, Mark Gordian Adam, Kai Thorsten Laser, Karl-Otto Dubowy, Andreas Entenmann, Daniela Karall, Ralf Geiger, Manuela Zlamy, Sabine Scholl-Bürgi

**Affiliations:** 1 Department of Pediatrics III, Division of Pediatric Cardiology, Medical University of Innsbruck, Anichstraße 35, 6020 Innsbruck, Austria; 2 Department of Pediatrics I, Division of Pediatric Cardiology, Medical University of Innsbruck, Anichstraße 35, 6020 Innsbruck, Austria; 3 Management Center Innsbruck, Department of Food Technologies, Maximilianstraße 2, 6020 Innsbruck, Austria; 4 BIOCRATES Life Sciences AG, Eduard-Bodem-Gasse 8, 6020 Innsbruck, Austria; 5 Center of Pediatric Cardiology and Congenital Heart Disease, Heart and Diabetes Center North Rhine-Westphalia, Ruhr-University of Bochum, Georgstraße 11, 32545 Bad Oeynhausen, Germany

**Keywords:** Amino acid metabolism, Congenital heart disease, Fontan, Metabolomics, Pattern recognition, Tandem mass spectrometry

## Abstract

**Introduction:**

Metabolomics studies are not routine when quantifying amino acids (AA) in congenital heart disease (CHD).

**Objectives:**

Comparative analysis of 24 AA in serum by traditional high-performance liquid chromatography (HPLC) based on ion exchange and ninhydrin derivatisation followed by photometry (*PM*) with ultra-high-performance liquid chromatography and phenylisothiocyanate derivatisation followed by tandem mass spectrometry (*TMS*); interpretation of findings in CHD patients and controls.

**Methods:**

*PM*: Sample analysis as above (total run time, ~ 119 min). *TMS*: Sample analysis by AbsoluteIDQ® p180 kit assay (BIOCRATES Life Sciences AG, Innsbruck, Austria), which employs PITC derivatisation; separation of analytes on a Waters Acquity UHPLC BEH18 C18 reversed-phase column, using water and acetonitrile with 0.1% formic acid as the mobile phases; and quantification on a Triple-Stage Quadrupole tandem mass spectrometer (Thermo Fisher Scientific, Waltham, MA) with electrospray ionisation in the presence of internal standards (total run time, ~ 8 min). Calculation of coefficients of variation (CV) (for precision), intra- and interday accuracies, limits of detection (LOD), limits of quantification (LOQ), and mean concentrations.

**Results:**

Both methods yielded acceptable results with regard to precision (CV < 10% *PM*, < 20% *TMS*), accuracies (< 10% *PM,* < 34% *TMS*), LOD, and LOQ. For both Fontan patients and controls AA concentrations differed significantly between methods, but patterns yielded overall were parallel.

**Conclusion:**

Serum AA concentrations differ with analytical methods but both methods are suitable for AA pattern recognition. *TM*S is a time-saving alternative to traditional *PM* under physiological conditions as well as in patients with CHD.

**Trial registration number:**

ClinicalTrials.gov Identifier NCT03886935, date of registration March 27th, 2019 (retrospectively registered).

**Supplementary Information:**

The online version contains supplementary material available at 10.1007/s11306-020-01741-8.

## Introduction

Metabolomics, the study of small organic molecules, their synthesis, and their breakdown, has identified in adult biventricular patients novel candidate biomarkers for congestive heart failure and vascular perturbations in addition to natriuretic peptides and troponins (Wang et al. 2018). Serum amino acid (AA) determinations in patients with normal cardiovascular anatomy and chronic heart failure have diagnostic and prognostic potential (Wang et al. 2018). To identify biomarkers for ventricular or circulatory failure in patients with complex congenital heart disease (CHD) is thus of growing interest. Especially in patients with single-ventricle types of CHD and Fontan circulation, traditional markers such as serum concentrations of N-terminal prohormone of brain natriuretic peptide are of limited use for non-invasive diagnostics and monitoring (Giannakoulas et al. 2010; Larsson et al. 2007). Exploration of the metabolic status of such patients also is under study: Fontan patients’ glucose and lipid metabolism is demonstratedly abnormal, but the metabolism of AA in such patients is largely unexplored (Ohuchi et al. 2009; Whiteside et al. 2013, 2016; Zyblewski et al. 2012; Raedle-Hurst et al. 2017).

Serum concentrations of AA are traditionally measured by high-performance liquid chromatography (HPLC) and ninhydrin derivatisation followed by photometry (*PM).* Post-column derivatisation enhances detectability of amino acids. This method is rather time-consuming (Deng et al. 2016). Metabolomic studies employ liquid chromatography tandem mass spectrometry (*TMS*), a method considered fast and easy-to-perform that is finding increased clinical application (Wang et al. 2018; Chong et al. 2018). *TMS* is often combined with pre-column derivatisation (as with phenylisothiocyanate [PTIC]), to improve reversed-phase separation and resolution. Additionally, since PTIC-derivatised amino acids are volatile, MS detection can be used. Alternatively, an ion-pairing agent can enhance separation on a reversed-phase column, speeding analysis even more by omitting pre-column derivatisation (Gu et al. 2007). A drawback of this method, however, is that MS spectra will show a higher than normal background due to the use of ion-pairing agents, rendering detection less sensitive (Ferre et al. 2019).

*TMS* might be an interesting alternative for AA measurements in patients with complex CHD. Measurement of serum AA is not now routinely performed in such patients, but initial metabolomic results are promising, as in the observation that altered serum concentrations of AA point towards inflammation and oxidative stress (Michel et al. 2020a, b).

For validation purposes, in this study we thus compared traditional *PM* with ultra-high-performance liquid chromatography (UHPLC) using PITC derivatisation followed by tandem mass spectrometry for analysis of 24 AA and related compounds in sera of Fontan patients and of healthy controls.

## Materials and methods

### Patients

At the Center of Pediatric Cardiology and Congenital Heart Disease, Heart and Diabetes Center North Rhine-Westphalia, Ruhr-University of Bochum, Germany, we prospectively examined both adult Fontan patients with a dominant left ventricle and age- and sex-matched healthy biventricular controls. Inclusion and exclusion criteria are described elsewhere (Michel et al. 2020a, b). Age, sex, weight, body mass index, vital parameters, cardiac risk factors, history of cardiac disease, and cardiac medication were assessed and blood was drawn for biochemical profiling during an outpatient-clinic visit (Michel et al. 2020a, b). The fasting patients underwent phlebotomy while recumbent. Determinations of analytes within our study required 0.5 mL of blood beyond the volume drawn for routine assessment.

### Preanalytics

The blood sample was directly drawn into a tube containing a clotting activator. To separate serum, within a maximum of 20 min after sample collection the sample was centrifuged (15 °C, 10 min, 2500 rcf). Serum aliquots were immediately frozen and stored at − 80 °C for further analyses (maximum storage time 4 months). Frozen samples were transported on dry ice to the analysing laboratories. Serum samples were thawed on ice and centrifuged immediately before analysis of the supernatant.

*PM.* Serum samples were deproteinised by acid hydrolysis using 6 M hydrochloric acid prior (Cunniff 1995). 500 µL aliquots were mixed with 1000 µL lithium citrate loading buffer (pH 2.2) (Beckman Coulter Diagnostics, La Habra, CA) and to 500 µL of this mixture were added 50 µL of S-2-aminoethyl-L-cysteine-hydrochloride internal standard (2.5 µmol/mL) and 50 µL of 50% sulfosalicylic acid. The mixture was vortexed and then centrifuged for 5 min at 10.880 rcf. The supernatant (300 µL) was mixed with 300 µL of lithium citrate loading buffer and 30 µL of 1 M sodium hydroxide solution. The mixture was stored at − 20 °C until analysis. 50 µL of the mixture described above were used for determination of AA concentrations. Analysis was performed using an automated AA analyser (Biochrom 30+ , Biochrom, Cambridge, UK) based on HPLC with an ion-exchange resin (Heinrikson and Meredith 1984). It was calibrated using an external standard. An internal standard served to monitor separation and derivatisation. A high pressure polyetheretherketone column (Laborservice Onken GmbH, Gründau, Germany) packed with Ultropac 8 cation exchange resin (LKB, Vienna, Austria) was used for separating AA. The ninhydrin = AA reaction yielded a colored complex used for photometric detection. Absorbance was measured at 440 nm (hydroxyproline, asparagine) and 570 nm (other analytes). Elution was conducted using lithium citrate regeneration buffer and lithium loading buffer in sequence. Total run time of the analysis, including column regeneration, was ~ 119 min.

*TMS*. The AbsoluteIDQ® p180 kit assay (BIOCRATES Life Sciences AG, Innsbruck, Austria) is a validated, commercially available targeted metabolomics assay that fits a variety of liquid chromatography mass spectrometry triple quadrupole instruments (Siskos et al. 2017). With this kit, a total of 188 metabolites from 6 compound classes can be analysed (AA, biogenic amines, acylcarnitines, glycerophospholipids, sphingolipids, and hexoses). It has already been used in many studies of human serum or plasma (Kühn et al. 2016; Ang et al. 2016; Schmidt et al. 2015). Measurement of AA and biogenic amines is based on PITC derivatisation, separation of analytes on a Waters Acquity UHPLC BEH18 C18 reversed-phase column (Waters, Vienna, Austria) using water and acetonitrile with 0.1% formic acid as mobile phases, and quantification on a Triple-Stage Quadrupole tandem mass spectrometer (Thermo Fisher Scientific, Waltham, MA) with electrospray ionisation in the presence of internal standards. The total run time is ~ 8 min per sample. Analyses are performed in 96-well-plate format, allowing measurement of batches of 80 samples at one time. The *TMS* instrument is calibrated periodically after each cleaning cycle, at least once a year, using Pierce™ Triple Quadrupole Calibration Solution (Thermo Fisher Scientific, Cat. No. 88325) according to manufacturer instructions, with necessary adjustments made automatically.

### Data processing

*PM* and *TMS* raw data were assembled in an Excel spreadsheet for further processing. For each QC sample, CV (for calculation of precision) and intra- and interday accuracies were calculated. Limit of detection (LOD) and limit of quantification (LOQ) were calculated for each metabolite. Mean concentrations and standard deviations were calculated for the metabolites.

### Measurement of precision, accuracy, LOD, LOQ

AA were quantified with commercially available physiological standard mixes for both methods (*PM*: ClinChek® Plasma Control, lyophilised, for Amino Acids (by Amino Acid Analyser), Level I, II, Recipe Chemicals + Instruments GmbH, Munich; *TMS*: Proprietary [BIOCRATES Life Sciences AG, Innsbruck, Austria]).

For *PM* using the standard mix, a serial dilution was performed confirming that all measured values were in the linear range of the assay. For *TMS*, instead of a serial dilution a traditional 7-point external standard calibration was performed for each AA showing that the measurement range was linear.

The inter-laboratory reproducibility of both assays was assessed by participating in ring trials (*PM*: ERNDIM (https://erndim.org*,* (Fowler et al. 2008)), *TMS*: (Siskos et al. 2017)). Additionally, assay accuracy and precision were determined for both methods individually by repeat measurements of the physiological AA standards.

Last, LOD and LOQ were determined for both assays individually. For *PM*, LOD and LLOQ were manually determined in the course of the serial dilution of the standard mix (the ULOQ was not determined for this method). For TMS, the LOD were determined by tripling the median of 3 blank measurements, and lower and upper LOQ (LLOQ, ULOQ) were set as the values of the lowest and highest calibrator for each assay, respectively (Siskos et al. 2017; Di Guida et al. 2016).

### Data preparation and statistical analysis

For *TMS*, to exclude metabolites with concentrations below the LOD, the raw data (µmol/L) were cleaned applying a modified 80% rule; thus, for statistical analysis, at least 80% valid values above LOD needed to be available per analyte in the samples for both groups (patients and controls). Remaining values below LOD were imputed applying a logspline method with values between LOD and LOD/2 (R package “logspline”); 9 values were imputed in total. This reduced the dataset to 143 analytes of *TMS*-measurements (30 AA or related compounds, 24 of which had a counterpart in *PM*).

### Analytes of *PM*-measurements were above LOD in all cases

After log transformation to approximate a normal distribution, the data set was used for univariate statistical analyses. Student’s two-sided dependent t-tests with a Benjamini–Hochberg correction identified significant differences between analyte values obtained using the two methods for both Fontan patients and controls (Benjamini and Hochberg 1995). P-values were calculated considering a p < 0.05 as statistically significant. An F-test with a Benjamini–Hochberg correction showed no significant difference between the variances.

To compare the methods, averages of the differences between the paired, log-transformed data from both were calculated and Bland–Altman plots were generated. 95% limits of agreement (LOA) were calculated for analyte values in Fontan patients and in control patients. Statistical analysis was done using the software package R (version 3.5.1., The R Foundation for Statistical Computing, Vienna, Austria) (R Core Team 2019).

## Results

Among the 398 Fontan patients seen in our outpatient clinic, only those with a dominant left ventricle were included (n = 176). After exclusion of all patients < 18 years of age (n = 105) and after applying all further exclusion criteria, 20 patients and their matched controls were left for analysis (Michel et al. 2020a, b). Serum concentrations of 24 AA were compared.

### Precision, accuracy, LOD, LOQ

Intraassay and interassay CV and accuracies for each analyte by method are given in Table 1. Both methods yielded acceptable results in respect of precision, with a CV of < 10% for *PM* (except for asparagine, hydroxyproline, and tryptophan) and of < 20% for *TMS* for all AA and with accuracies < 10% for *PM* and < 34% for *TMS* (except for tryptophan and hydroxyproline). Values for tryptophan (*PM*) showed a slight downward slope in intra-day measurements. LOD, LLOQ, and ULOQ-values for each individual analyte are given in Supplemental Table 1. The range for *PM*-derived LOD was 1.1–14.8 µmol/L and the range for *PM*-derived LLOQ was 0.8–29.5 µmol/L. The range for *TMS*-derived LOD was 0.1–160 µmol/L and the ranges for *TMS*-derived LLOQ and ULOQ were 1–160 µmol/L and 80–1600 µmol/L, respectively.

**Table 1:** Absolute serum analyte concentrations and p-values of comparisons of those concentrations in controls and in Fontan patients, as measured by both methods (PM and TMS)

Analyte [µmol/L]	Control PM	Control PM relative SD	Control TMS	Control TMS relative SD	Control PM *vs*. Control TMS, p-value	Fontan PM	Fontan PM relative SD	Fontan TMS	Fontan TSM relative SD	Fontan PM *vs*. Fontan TMS, p-value
Alanine	386.2 ± 83.6	0.22	410.5 ± 119.9	0.30	0.3316	381.0 ± 75.0	0.20	401.4 ± 82.0	0.21	**0.0457**
Alpha-AAA	5.3 ± 3.0	0.38	0.77 ± 0.43	0.57	**6.2E−07**	3.9 ± 2.4	0.39	1.12 ± 0.6	0.55	**6.8E−07**
Arginine	95.7 ± 18.1	0.19	108.3 ± 21.2	0.20	**0.0038**	86.4 ± 29.9	0.35	91.7 ± 26.1	0.29	**0.0353**
Aspartic acid	16.2 ± 8.2	0.14	17.2 ± 6.8	0.16	**5.4E−07**	16.9 ± 9.3	0.19	18.1 ± 10.2	0.18	**1.5E−10**
Asparagine	65.8 ± 9.4	0.42	50.7 ± 7.8	0.40	0.5224	56.4 ± 10.8	0.55	43.7 ± 7.5	0.58	0.7544
Citrulline	33.8 ± 6.8	0.20	29.2 ± 6.4	0.23	**6.3E-05**	33.4 ± 7.6	0.23	29.4 ± 6.1	0.21	**0.0002**
Glutamine	583.0 ± 69.8	0.12	749.0 ± 108.9	0.15	**3.4E−07**	565.1 ± 98.9	0.18	717.4 ± 130.7	0.19	**3.4E−07**
Glutamic acid	42.3 ± 18.9	0.45	46.9 ± 31.3	0.46	0.5036	83.4 ± 65.9	0.79	85.9 ± 61.8	0.74	0.2565
Glycine	251.4 ± 51.2	0.20	345.3 ± 89.4	0.27	**3.7E−08**	243.2 ± 47.3	0.19	321.6 ± 72.2	0.23	**4.7E−08**
Histidine	93.2 ± 10.6	0.11	110.4 ± 23.8	0.22	**0.0033**	81.1 ± 12.6	0.16	93.08 ± 14.6	0.16	**0.0013**
t4-OH-Pro	19.2 ± 13.2	0.61	11.7 ± 5.1	0.45	**0.0822**	20.9 ± 12.2	0.52	15.8 ± 4.9	0.32	**0.0438**
Isoleucine	64.9 ± 18.2	0.28	100.9 ± 38.5	0.39	**4.8E−06**	69.7 ± 19.2	0.28	106.6 ± 30.8	0.30	**6.5E−10**
Leucine	135.6 ± 31.1	0.23	232.8 ± 103.9	0.46	**3.4E−07**	131.2 ± 34.5	0.26	222.9 ± 76.6	0.35	**1.5E−10**
Lysine	178.2 ± 29.2	0.16	165.8 ± 31.6	0.20	**0.0080**	179.5 ± 29.8	0.17	168.1 ± 22.9	0.14	**0.0147**
Methionine	28.9 ± 6.0	0.21	29.4 ± 7.6	0.27	0.9212	27.8 ± 8.3	0.30	27.9 ± 8.1	0.30	0.9733
Ornithine	86.7 ± 22.6	0.26	111.6 ± 39.9	0.37	**2.2E−05**	103.3 ± 34.6	0.33	139.4 ± 80.2	0.59	**0.0003**
Phenylalanine	68.1 ± 10.9	0.16	76.9 ± 12.5	0.17	**0.0085**	69.3 ± 11.8	0.17	77.7 ± 16.5	0.22	**0.0029**
Proline	235.2 ± 58.5	0.25	265.7 ± 60.1	0.23	**0.0005**	226.0 ± 56.0	0.25	259.4 ± 62.1	0.25	**1.3E−05**
Serine	132.0 ± 19.5	0.15	146.5 ± 23.1	0.16	**0.0129**	136.6 ± 21.2	0.16	158.4 ± 32.3	0.21	**0.0005**
Taurine	141.2 ± 50.6	0.36	132.6 ± 55.1	0.43	0.2413	109 ± 51.5	0.47	100.4 ± 55.7	0.57	**0.0002**
Threonine	148.3 ± 30.4	0.21	127.3 ± 27.7	0.22	**1.3E−04**	132.6 ± 26.4	0.20	105.6 ± 20.5	0.20	**0.0000**
Tryptophane	86.4 ± 22.1	0.26	89.5 ± 22.7	0.26	0.5899	89.5 ± 22.4	0.25	90.5 ± 19.7	0.22	0.7814
Tyrosine	78.3 ± 22.5	0.20	83.6 ± 18.9	0.23	0.4115	77.5 ± 19.5	0.25	87.0 ± 22.5	0.26	**0.0005**
Valine	254.2 ± 49.0	0.19	290.7 ± 74.9	0.26	**0.0193**	257.8 ± 54.6	0.21	298.9 ± 70.6	0.24	**0.0002**

Serum values of controls and Fontan patients are given as mean ± standard deviation (relative standard deviation). p < 0.05 is printed in bold letters. Alpha-AAA, alpha-aminoadipic acid; t4-OH-Pro, trans-4-hydroxyproline

**Fig. 1:**
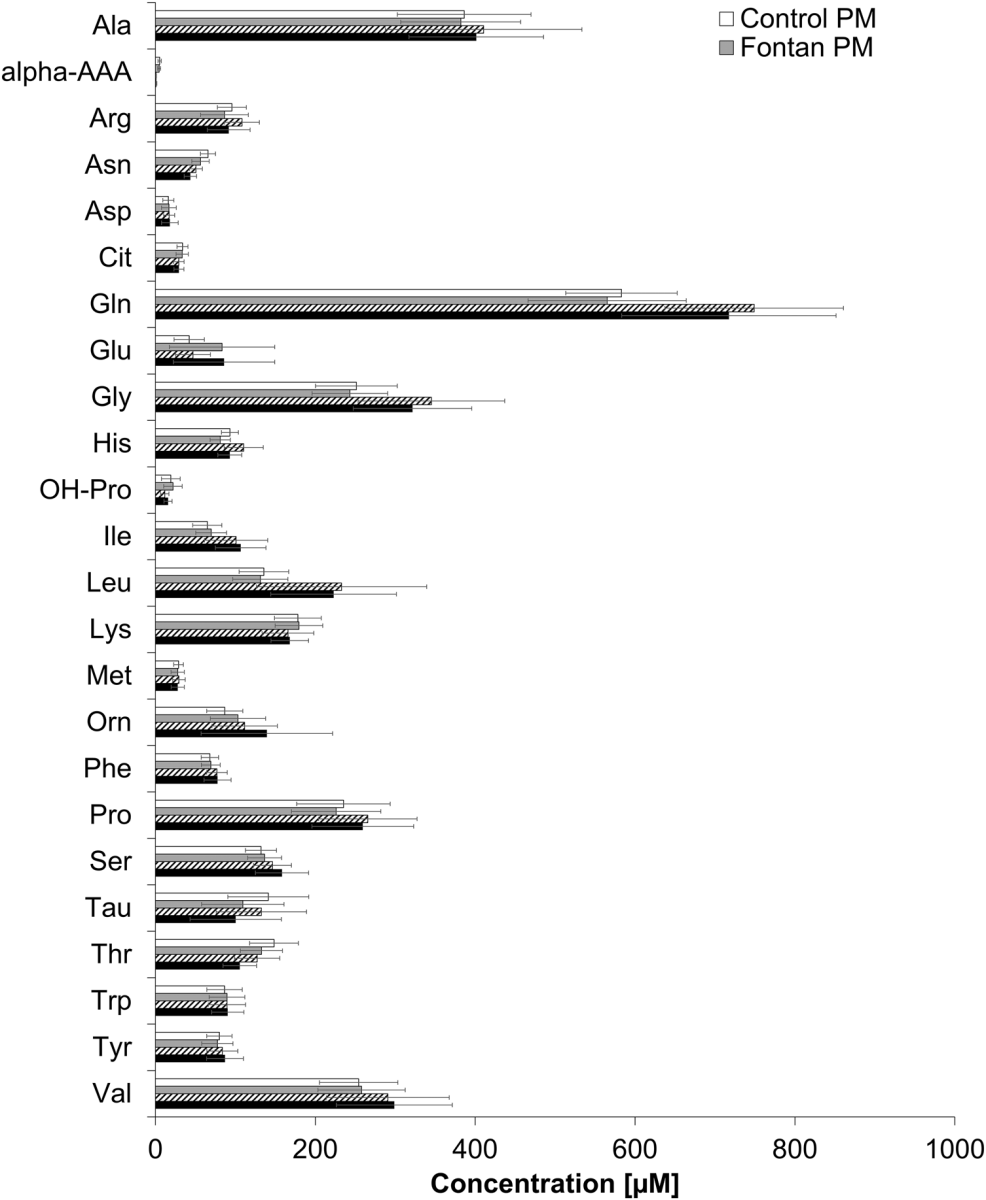
Amino acid serum concentrations (means and standard deviations) by client group (Fontan *vs*. control) and method (*PM vs. TMS*). White boxes: controls, *PM*; white boxes with black stripes: controls, *TMS*; grey boxes: Fontan patients, *PM*; black boxes: Fontan patients, *TMS*. Ala, alanine; alpha.AAA, alpha-aminoadipic acid; Arg, arginine; Asn, asparagine; Asp, aspartic acid; Cit, citrulline; Gln, glutamine; Glu, glutamic acid; Gly, glycine; His, histidine; OH-Pro, hydroxyproline; Ile, isoleucine; Leu, leucine; Lys, lysine; Met, methionine; Orn, ornithine; Phe, phenylalanine; Pro, proline; Ser, serine; Tau, taurine; Thr, threonine; Trp, tryptophan; Tyr, tyrosine; Val, valine

Details of differences in AA concentrations between control and Fontan patients as determined by *TMS* are reported elsewhere (Michel et al. 2020a, b). In both control and Fontan patients, mean analyte concentrations determined by *PM* versus *TMS* differed (p-values, Table 1). In control samples, for 17 of the 24 metabolites the measured concentrations measured with *PM* differed significantly from those measured with *TMS*, yielding higher *TMS*-measured concentrations for 12 analytes and lower concentrations for 5 analytes. In Fontan samples, the measured concentrations differed significantly between the two methods for 20 of the 24 metabolites, yielding higher *TMS*-measured concentrations for 14 analytes and lower for 6 analytes (Fig. 1, Table 1). Despite the significant concentration differences, the overall patterns were similar for control patients regardless of analytic method, and the overall patterns also were similar for the Fontan patients regardless of analytic method (Fig. 1).

**Fig. 2:**
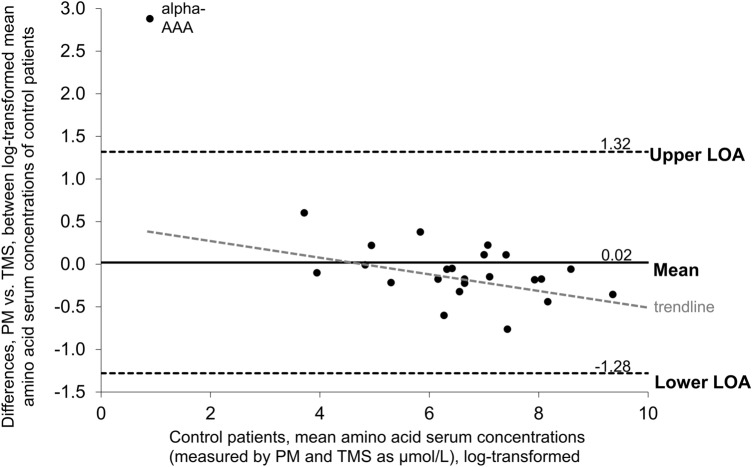
Bland–Altman-plot comparing serum concentrations of analytes in Fontan patients determined by *PM* with those determined by *TMS*. Diff, difference; LOA, limit of agreement. LOA indicates 95% LOA

**Fig. 3:**
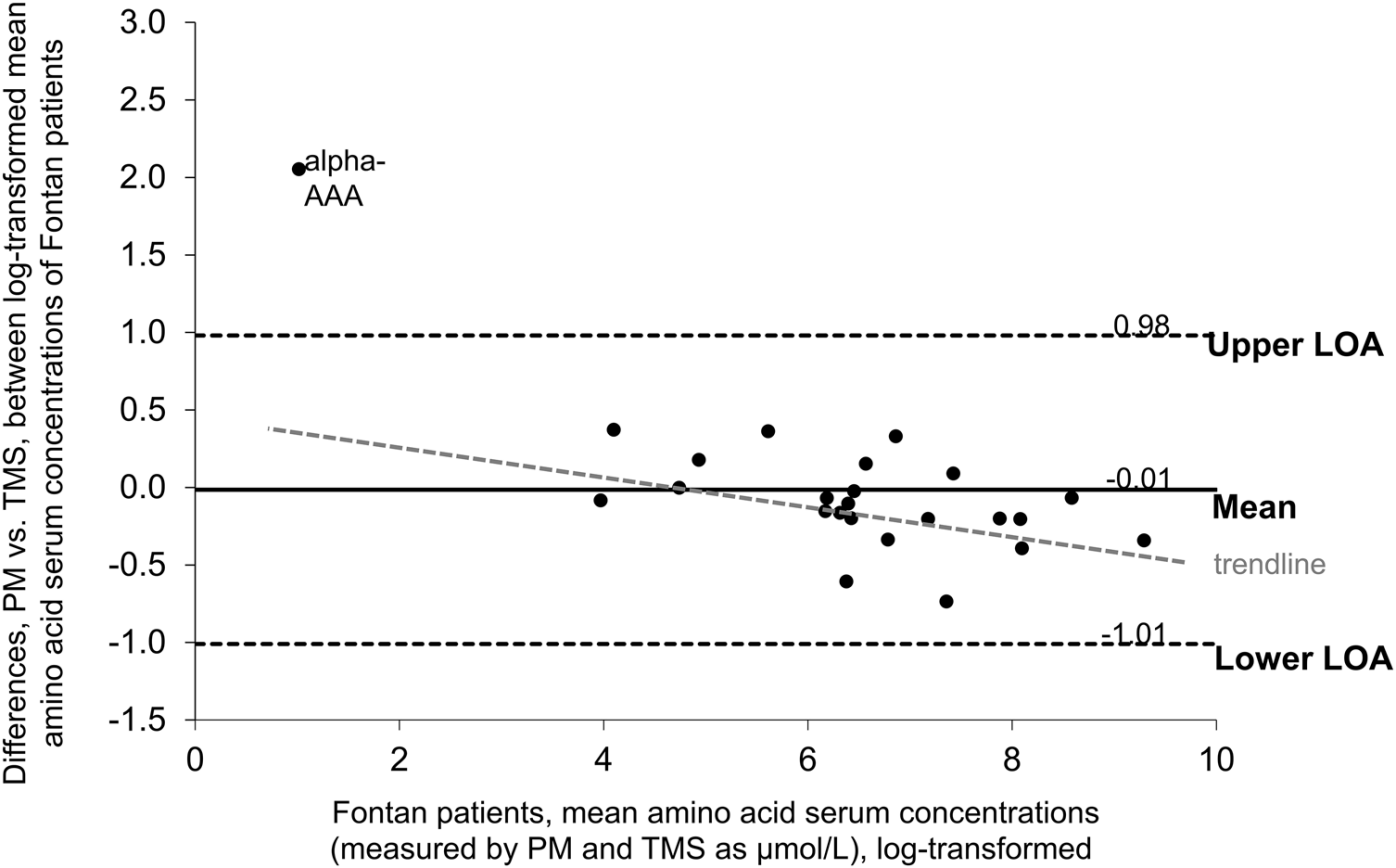
Bland–Altman-plot comparing serum concentrations of analytes in controls determined by *PM* with those determined by *TMS*. Diff, difference; LOA, limit of agreement. LOA indicates 95% LOA

The average differences between the paired log-transformed data for the two methods were 0.02 for controls and − 0.01 for Fontan patients. Being close to 0, these values indicate absence of systematic bias introduced by the *TMS* method (with *PM* as standard). The differences between concentrations determined by *PM* or *TMS* were graphed separately for controls and patients against the mean of the measurements of the two different methods as Bland–Altman plots (Figs. 2 and 3). Though the metabolites clustered a bit more in the Fontan data plot, resulting in a smaller range bordered by the LOA, the patterns overall otherwise were very similar, indicating that differences between the analytical methods did not differently affect results from controls and results from Fontan patients.

All metabolites’ concentrations were within 95% LOA except for alpha-aminoadipic acid, which on graphing appears as an outlier (Figs. 2 and 3). This could be related to the fact that alpha-aminoadipic acid concentrations in the samples in this study were near the detection limit, with 15% and 30% of the *TMS* values being below LOD from Fontan patients and controls, respectively.

The plots reveal a negative trend of differences that is proportional to the magnitude of the measurement. The trend is illustrated with a trendline in Figs. 2 and 3 (alpha-aminoacidic acid, as an outlier, was excluded from trendline calculations). The trendlines’ negative slopes indicate that *TMS* tends to yield values lower than those obtained with *PM* if analyte concentrations are low, and values higher than those obtained with *PM* if analyte concentrations are high. The LOA thresholds correspond to the expected differences due to employing the one or the other method. These differences are likely to be significant for metabolites with low concentrations, but irrelevant for metabolites with high concentrations, depending on the relative impact upon the difference from the corresponding concentration value.

## Discussion

This study is, we believe, the first inter-method assessment of a widely used targeted metabolomics platform based on UHPLC-tandem mass spectrometry and PITC derivatisation for human serum in a unique patient group with complex CHD palliated by the Fontan operation to yield a univentricular circulation, although untargeted profiling metabolomics have been compared, as have interlaboratory targeted profiling metabolomics (Siskos et al. 2017; Viant et al. 2009). The *TMS* equipment that we used has been employed in many studies of human serum and plasma (Fowler et al. 2008; Di Guida et al. 2016; Benjamini and Hochberg 1995), and UHPLC-based AA *TMS* has been approved for use in biventricular patients with heart failure (Wang et al. 2018; Hunter et al. 2016; Alexander et al. 2011; Tenori et al. 2013; Würtz et al. 2015). However, *TMS* has not before been deployed in patients with complex CHD.

The main finding of our study is that both *PM* and *TMS* yielded results acceptable in respect of precision and accuracy in the analysis of serum concentrations of AA in patients with complex CHD. In both patient and control cohorts, however, values for serum AA concentrations differed significantly between gold-standard *PM* results and *TMS* results*,* with *PM* in most cases generating lower numerical analyte values*.* Nevertheless, both methods yielded similar patterns of serum AA concentrations for patients and similar patterns of serum AA concentrations for controls.

Perhaps susceptibility to oxidation underlies high values for CV among *PM* measurements of serum concentrations of tryptophan: If some tryptophan was degraded during measurement, results might vary. The slight downward slope for tryptophan values found in intra-day measurement runs may support this hypothesis.

An explanation for the high values for CV and accuracy of *PM* measurements of the serum concentrations of hydroxyproline and asparagine may be that these AA are measured at 440 nm, whilst the other analytes are measured at 570 nm. At 440 nm the signal to noise ratio is lower, which means that the measurements are less precise.

Significant differences between results depending on method might be explained by differences in sample handling, with pre-column PITC derivatisation for the most part yielding slightly higher metabolite concentrations in serum samples than those seen with classical post-column ninhydrin derivatisation. Differences also might originate in use of slightly different measuring equipment (pipettes, centrifuge tubes, etc.). Whilst the *TMS* kit used was validated for all main mass spectrometry manufacturers (Sciex, Waters, Agilent), each mass spectrometer has its peculiarities (Siskos et al. 2017). Hence, although both methods performed very well with regard to assay performance, variability, LOD, and LOQ, not only method- but also equipment- and laboratory-specific reference values are mandatory. This is especially true for AA, for which reference values can vary substantially with age and diet (Haschke-Becher et al. 2016).

That the sensitivity of *TMS* is higher than that of *PM* as well as the higher selectivity of *TMS* might cause differences in metabolite values. *PM* permits measurement at only one wavelength at a time, which may be problematic for analytes detected at the same wavelength or at nearby wavelengths, whilst *TMS*, to avoid this crosstalk, permits choice among separate multiple reaction-monitoring transitions for similar molecules. *PM* also varied less interday than *TMS* (Table 1), another source for differences in results.

Whilst concentration results differed significantly between methods for most of the individual AA assessed in this study, Bland–Altman plots show that the methods are actually quite commensurable with no systematic bias present in the differences and a comparatively narrow agreement interval bordered by the LOA.

An advantage of *TMS* over *PM* is the difference in total run time: given that reference values are provided, *TMS* could greatly expedite analysis. This could be especially helpful for clinical use (Grebe and Singh 2011). Moreover, in contrast to *PM* which depends on sequential analysis and allows analysis of only a small number of analytes, *TMS* permits simultaneous analysis of a very high number (> 600) of analytes. However, *TMS* analyses are still very complex, requiring specially trained expert staff and maintenance service, and are more expensive per analyte than are *PM* analyses (Vogeser 2003).

### Clinical implications

This study demonstrated statistically significant method-dependent differences in results, implying that laboratory-specific and method-specific reference values are desirable when quantitating AA in serum. However, for both methods calculated analyte concentrations were in similar ranges, rendering choice of method clinically negligible. Noteworthy is that both methods were suitable for recognition of concentration patterns (Michel et al. 2020a, b).

We tried to minimise methodologic and clinical-factor bias by ensuring that only one person (MM) handled samples during initial processing, and that this processing was immediate (Anton et al. 2015); by studying only serum and not plasma (Yu et al. 2011); and by choosing age- and sex-matched controls (Floegel et al. 2011, 2014; Yu et al. 2012). Still, anthropometric features, smoking, the effects of sleep restriction and circadian clock disruption, as well as genome-wide heritable variation in human metabolism, are bias factors to keep in mind (Xu et al. 2013; Davies et al. 2014; Nicholson et al. 2011; Illig et al. 2010).

### Limitations of the method comparison

The precision of both methods, with an achievable CV of measurements of 5–15%, might be the most important limiting factor: even if an accuracy of < 15% is considered acceptable, both methods are to some extent inaccurate (European Commission 2002). In addition, when comparing methods, large numbers of patients and controls are desirable. This would require a multicenter approach.

## Conclusion

This is the first inter-method comparison in a unique patient group with Fontan hemodynamics and in healthy controls of a standard method of AA quantitation in human serum with a widely used targeted metabolomics platform based on UHPLC-tandem mass spectrometry and PITC derivatisation. Our work demonstrates that serum AA concentrations in this special patient group as detected by two different methods differ by method as well as by subject group, indicating that method-specific (and laboratory-specific) reference values must be established for patients with CHD and for healthy controls. Analysis of AA concentrations in Fontan patients using UHPLC-tandem mass spectrometry and PITC derivatisation qualifies as a time-saving alternative to the gold standard of HPLC-photometry and ninhydrin derivatisation. Even without specific reference values, both methods are suitable for serum AA concentrations pattern recognition., AA studies offer a promising approach for the early detection of organ and metabolic alterations in patients with complex CHD.

## Electronic supplementary material

Below is the link to the electronic supplementary material.

Electronic supplementary material 1 (XLSX 18 kb)

Electronic supplementary material 2 (XLSX 31 kb)

## Data Availability

For the database reported in this study, *cf*. Supplemental Table 2.

## Code availability (software application)

Proprietary (BIOCRATES Life Sciences AG, Innsbruck, Austria).
